# Controlling HIV Epidemics among Injection Drug Users: Eight Years of Cross-Border HIV Prevention Interventions in Vietnam and China

**DOI:** 10.1371/journal.pone.0043141

**Published:** 2012-08-27

**Authors:** Theodore M. Hammett, Don C. Des Jarlais, Ryan Kling, Binh Thanh Kieu, Janet M. McNicholl, Punneeporn Wasinrapee, J. Stephen McDougal, Wei Liu, Yi Chen, Donghua Meng, Ngu Doan, Tho Huu Nguyen, Quyen Ngoc Hoang, Tren Van Hoang

**Affiliations:** 1 Abt Associates Inc., Cambridge, Massachusetts, United States of America; 2 Beth Israel Medical Center, New York, New York, United States of America; 3 Abt Associates Inc., Hanoi, Vietnam; 4 DHAP, Laboratory Branch, Centers for Disease Control and Prevention, Atlanta, Georgia, United States of America; 5 Thai Ministry of Public Health-US CDC Collaboration, Nonthaburi, Thailand; 6 National Center for HIV/AIDS, STDs, and TB Prevention, Centers for Disease Control and Prevention, Atlanta, Georgia, United States of America; 7 Guangxi Center for Disease Control, Nanning, China; 8 Ning Ming County Health Department, Ning Ming City (Guangxi), China; 9 Abt Associates Inc., Ho Chi Minh City, Vietnam; 10 Lang Son Provincial HIV/AIDS Center, Lang Son City, Vietnam; 11 Ha Giang Provincial Health Services, Ha Giang City, Vietnam; Vanderbilt University, United States of America

## Abstract

**Introduction:**

HIV in Vietnam and Southern China is driven by injection drug use. We have implemented HIV prevention interventions for IDUs since 2002–2003 in Lang Son and Ha Giang Provinces, Vietnam and Ning Ming County (Guangxi), China.

**Methods:**

Interventions provide peer education and needle/syringe distribution. Evaluation employed serial cross-sectional surveys of IDUs 26 waves from 2002 to 2011, including interviews and HIV testing. Outcomes were HIV risk behaviors, HIV prevalence and incidence. HIV incidence estimation used two methods: 1) among new injectors from prevalence data; and 2) a capture enzyme immunoassay (BED testing) on all HIV+ samples.

**Results:**

We found significant declines in drug-related risk behaviors and sharp reductions in HIV prevalence among IDUs (Lang Son from 46% to 23% [p<0.001], Ning Ming: from 17% to 11% [p = 0.003], and Ha Giang: from 51% to 18% [p<0.001]), reductions not experienced in other provinces without such interventions. There were significant declines in HIV incidence to low levels among new injectors through 36–48 months, then some rebound, particularly in Ning Ming, but BED-based estimates revealed significant reductions in incidence through 96 months.

**Discussion:**

This is one of the longest studies of HIV prevention among IDUs in Asia. The rebound in incidence among new injectors may reflect sexual transmission. BED-based estimates may overstate incidence (because of false-recent results in patients with long-term infection or on ARV treatment) but adjustment for false-recent results and survey responses on duration of infection generally confirm BED-based incidence trends. Combined trends from the two estimation methods show sharp declines in incidence to low levels. The significant downward trends in all primary outcome measures indicate that the Cross-Border interventions played an important role in bringing HIV epidemics among IDUs under control. The Cross-Border project offers a model of HIV prevention for IDUs that should be considered for large-scale replication.

## Introduction

Almost 150 countries on all continents had reported injection drug use by 2007 and 120 of these had reported related HIV infections. HIV prevalence among the 16 million injection drug users (IDUs) worldwide is estimated at 19% [1]. Injection drug use is driving HIV epidemics in many countries in Asia, the Middle East, and Eastern Europe [1]. This is true in southern China and all of Vietnam. Controlling the HIV/AIDS epidemics in these countries depends on controlling HIV among IDUs [2].

There are more than 200,000 IDUs in Vietnam (representing about 0.7% of the male population aged 15–65), with an overall HIV prevalence of 30% (as high as 56% in some provinces); approximately 60% of all reported HIV cases have been among IDUs [3] (National Institute of Hygiene and Epidemiology and FHI360, unpublished data, 2009). In China, there are between 1.8 and 3 million IDUs (0.4%–0.6% of the male population aged 15–64), with national HIV prevalence estimated at 8%–19% [1]. Prevalence is higher than 50% in several provinces and about 32% of HIV infections are attributed to injection drug use [4].

The effectiveness of interventions for controlling HIV epidemics among IDUs –peer outreach, needle/syringe provision programs (NSP) and opioid substitution treatment (OST) – is supported by many research studies [5]. Epidemic modeling has shown that combination prevention composed of NSP, OST, and antiretroviral treatment will significantly reduce HIV incidence among IDUs [6], [7].

There have been NSPs in both China and Vietnam since the late 1990s. In China, small pilots gave way to a larger government-funded program with more than 1,000 sites across the country, although coverage remains quite low [8]. In Vietnam, there were also a few pilots in the late 1990s and some expansion of NSP since then, but these programs are still predominantly funded by international donors. The government has not yet assumed responsibility for scaling up NSP in Vietnam. While the Vietnamese and Chinese governments have supported harm reduction interventions, both countries still maintain important elements of a “social evils” approach toward drug use, which has resulted in large-scale arrest and commitment of IDUs to drug detention centers [9]–[13].

To counter the argument that confinement of drug users is the best way to contain HIV, further evidence of the effectiveness of community-based harm reduction such as NSP and OST is needed. There have been only a few other evaluations of NSP in Vietnam and China and these relied on quite limited data [14]–[15]. This paper presents a long-term evaluation of the Cross-Border HIV Prevention Project for IDUs in Southern China and Northern Vietnam. It is based on analysis of perhaps the longest data series on HIV among IDUs in Asia, assembled from cross-sectional surveys of IDUs carried out between 2002 and 2011 [16]–[19]. We focus here on trends in primary outcomes: HIV risk behaviors, HIV prevalence, and HIV incidence.

The idea for the Cross-Border Project originated at a training session on HIV prevention for IDUs in Kunming, China in 1997. During that meeting, two co-authors of the present paper (T.H., D.D.J.) discussed with counterparts from China and Vietnam the possibility of developing a harm reduction intervention for IDUs in a China-Vietnam border region experiencing linked epidemics of heroin injection and HIV infection. After more than four years of work to negotiate the necessary partnerships and to assemble the required funding, the Cross-Border Project’s interventions for IDUs were launched in Ning Ming County (Guangxi), China in June, 2002 and in Lang Son Province, Vietnam in September, 2002. In 2003, interventions for IDUs began in Ha Giang Town, Vietnam.

## Methods

### Interventions

**Figure 1:**
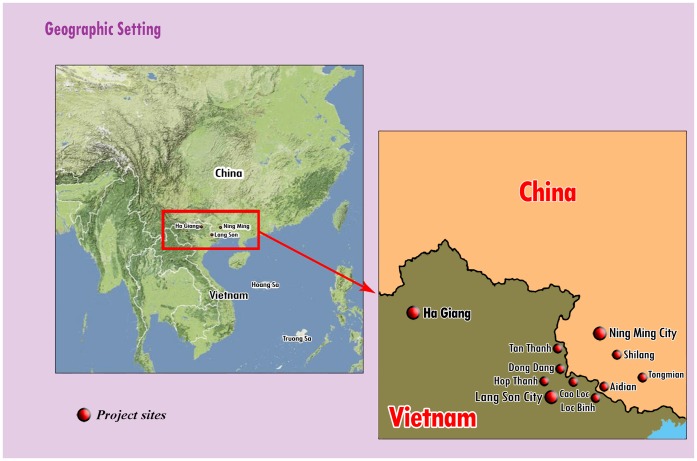
Map of Cross-Border Project sites.

The Cross-Border Project was the first HIV prevention project for IDUs in which the same interventions were implemented on both sides of an international border, in this case the border between northern Vietnam and southern China. Beginning in the early to middle 1990s, this area experienced epidemics of heroin injection and subsequently HIV infection among IDUs. As shown by Beyrer et al. [20], an important heroin trans-shipment route ran from the Golden Triangle of Laos, Thailand, and Burma into Northern Vietnam and thence into Southern China and to Hong Kong and the larger world. As heroin was shipped through the border region, drug users shifted from traditional opium smoking to heroin smoking and then quite quickly, for reasons of cost-effectiveness, to heroin injection. A map of the Cross-Border Project sites is presented in Figure 1.

As we have shown in an earlier paper, there was a gradient of HIV prevalence in the Cross-Border sites from the highest in the localities in Vietnam farthest from the China border to the lowest in the Chinese sites farthest from the Vietnam border [21]. While other patterns of HIV transmission may also have occurred, this prevalence gradient suggests that the dual epidemics of heroin injection and HIV infection moved from south to north through the border area. We also showed that border crossing behavior was associated with being HIV-infected among IDUS from both Chinese and Vietnamese sites [22].

The Cross-Border interventions are based on a “peer outreach” model developed in the U.S. in the late 1980s [23]–[26]. This model was implemented from the beginning of the project in 2002 in Lang Son Province, Vietnam and Ning Ming County, China. In Ha Giang Town between 2003 and 2005, a “peer-driven intervention” (PDI) was implemented. This model, which essentially applies Respondent-Driven Sampling to delivery of HIV prevention messages and commodities, involves large numbers of IDUs as recruiters and deliverers of harm reduction services in return for small rewards [27], as opposed to the peer outreach model, which employs regular peer educators who receive monthly stipends for their work. In 2005, the PDI in Ha Giang Town was converted to a peer outreach model because we concluded that it had already reached all of the IDUs – indeed, the PDI reached 900 IDUs, far more than the 700 estimated by the health department to live in Ha Giang.

In the peer outreach model, initial groups of peer educators (PEs) were recruited by local health departments based on performance during initial training, recommendations from IDU “gatekeepers”, and expressed interest in joining the project. The original intention was that the peer educators be former IDUs. However, it quickly became apparent that very few IDUs in these sites had been able to stop injecting heroin. Therefore, it was necessary to recruit primarily current IDUs. Most of the PEs are young men but women PEs have been recruited to reach women IDUs as well as sexual partners of IDUs and sex workers in companion interventions for women at risk.

The initial groups of PEs received two weeks of training and the local health departments have provided periodic refresher training. In addition, the peer educators’ weekly meetings include training on special topics. The peer educators are supervised locally by health department staff. Because there were different funding sources and local cost norms, PEs’ stipends differed across sites: from $30 per month in Lang Son to $46 in Ha Giang to $117 in Ning Ming.

For a variety of reasons, mostly associated with the peer educators’ ongoing drug use, commitment to drug detention centers, arrest for petty crimes, or moving away, there has been substantial turnover among them. Turnover rates varied by year and by site but sometimes reached 50% per year. Local health department staff recruit and train replacements as needed.

The peer educators regularly contact other IDUs in the community and provide them with information on reducing drug use- and sex-related HIV risks, verbally and through distribution of brochures. They distribute new needles/syringes, ampoules of sterile water for injection solution, and condoms and vouchers redeemable for these items at participating pharmacies. The peer educators also perform a valuable public health service by collecting and disposing of used needles/syringes that might otherwise put community residents at risk.

The average annual cost of these interventions was $60,000–$75,000 per province (that is, all sites in the province), including the cross-sectional surveys. (More detailed information on the interventions may be found in a replication manual available from the corresponding author [28]).

The interventions aim to reduce HIV risk behaviors among IDUs, thereby stabilizing HIV prevalence and reducing HIV incidence on both sides of the border and preventing cross-border HIV transmission. We consider this a structural intervention because it seeks not only to influence the behavior of individual IDUs (e.g., reduction or elimination of the sharing of injection equipment) but also to diffuse HIV risk reduction messages and sterile injection equipment more widely through the IDU community [29]–[30].

The Cross-Border interventions have reached substantial proportions of the IDUs in the project sites through direct contact with PEs and “secondary exchange” in which IDUs receiving needles/syringes directly from the project pass on some of these to other IDUs. Through this and other means, the HIV risk reduction information and safer injection materials disseminated by the project are also reaching IDUs who do not personally interact with the project.

The cross-border project also seeks to diffuse knowledge of HIV and build support for the interventions in the general community. Project staff have regular meetings with police, local government bodies (e.g., People’s Committees), Communist Party officials, and mass organizations (e.g., Women’s Union, Youth Union). Staff and peer educators also regularly attend community meetings. These interactions build and maintain support for the interventions. Government leaders in Ning Ming, Lang Son, and Ha Giang gave initial approval to the interventions and directed all departments, including the police, as well as the general community, to give their support and cooperation. This support has been maintained but it requires constant nurturing through regular meetings and other interactions. Peer educators and project staff must repeatedly explain that the purpose of the project is to prevent disease and not to encourage drug use or give special treatment to drug users. Misinformation and misunderstanding are seemingly endemic, and the peer educators and project staff must constantly work to prevent misinformation and misunderstanding from undermining the interventions [28].

### Overall Evaluation Design

The evaluation of structural interventions poses complex methodological challenges. We believe that our interventions are appropriately evaluated at the community level rather than the individual level. Randomized controlled trials of these interventions pose serious ethical and cost issues.

Our design relies primarily on serial cross-sectional surveys of IDUs (including behavioral interviews and HIV testing) just before the start of the interventions and at follow up intervals thereafter. All survey waves reported on in this paper were carried out in the same sites in Ning Ming County, China (Ning Ming City, Aidian, Shilang, and Tongmian) and Lang Son (Lang Son City, Cao Loc, Loc Binh, Hop Thanh, Dong Dang, and Tan Thanh) and Ha Giang Provinces (Ha Giang Town), Vietnam. Data collection methods were largely parallel in Ning Ming County, Lang Son and Ha Giang, with some variation in the strategies used to recruit IDUs for the surveys. Routine monitoring data were employed to gauge levels of activities and commodities provided.

### Subject Recruitment

To be eligible, survey participants had to be at least 18 years of age and to have injected heroin in the past 6 months. In all sites, modified “snowball” techniques were used. These involved identification of initial respondents through project PEs, lists of registered IDUs, and/or IDUs present at mapped “shooting” places or other gathering places, with the remainder of sample quotas from each site recruited through snowball or chain referral methods. Chinese participants received 20 yuan (approximately $2.50) for the interview and blood sample, 5 yuan for each additional male respondent recruited, and 10 yuan for each additional woman respondent recruited. Vietnamese participants received 30,000 dong (about US$1.50) for the interview and blood sample.

### Informed Consent

An interviewer explained the survey procedures to each prospective participant. In Vietnam, a verbal informed consent was obtained from participants, with the interviewer certifying that consent by signing the form. This procedure was recommended by the Institutional Review Board of the National AIDS Standing Bureau in order to provide more assurance of confidentiality to prospective participants. In China, a standard signed informed consent was obtained from each participant.

Unique ID codes were constructed for each IDU participant composed of several letters and numbers representing, for example, the first letter of the mother’s family name and the subject’s date of birth. The objective was to have a unique identifier composed of items that participants could readily reconstruct in order to obtain their HIV test results. Test results were coded only by these identifiers.

### Questionnaire

A structured instrument was used for the interviews, based on version 2b of the questionnaire employed in the World Health Organization’s Drug Injection Study, Phase II [31]. The questionnaire includes a series of demographic and self-reported behavioral items. Most of the behavioral questions pertain to specific drug-related and sexual activities and measures of participation in the intervention during the 6-month period prior to the interview. (The full questionnaire is available upon request from the corresponding author.).

The interviews were conducted by trained staff of the local health departments. The baseline IDU survey in Lang Son Province, Vietnam was conducted in January, 2002 with 7 subsequent waves between March, 2003 and October, 2009. The 36-month and 60-month surveys had to be skipped because of temporary gaps in funding. In Ha Giang Town, Vietnam the baseline survey took place in January, 2003 with 9 additional waves between August, 2003 and February, 2011. The baseline survey in Ning Ming County, China occurred in July-August, 2002 with 7 subsequent waves between April, 2003 and June, 2008. Thus, in total we have data from 26 cross-sectional survey waves among IDUs across all sites.

### HIV Testing

The surveys included HIV antibody testing. Participants were given pre- and post-test counseling at local health centers. Blood was drawn by trained phlebotomists at the time of the interviews. Participants were told to return to the local health center on a certain date to receive their test results using their ID numbers.

In China, testing was by double ELISA (Vironostika HIV-uni-form, Organon [Holland]) with confirmation of initial HIV-positive results by Western blot methodology (Genelabs Diagnostics). All testing was conducted at the laboratory of the Guangxi Center for HIV/AIDS Prevention and Control in Nanning. In Vietnam, testing was performed at the laboratory of the Lang Son and Ha Giang Provincial Health Services using the Serodia SFD screening test (Biorad [France]) and double ELISA (Genescreen, Biorad [France]; Vironostika, Organon [Holland]). This is the official protocol of the Vietnam Ministry of Health.

### BED Testing

To identify recent infections and inform HIV incidence estimates, we conducted BED capture-enzyme immunoassay testing [32]–[34] on HIV-positive serum/plasma samples using the Calypte HIV-1 BED EIA (Calypte Biomedical Corporation, Rockville, MD) for all Ning Ming samples, Lang Son 48-, 72-, and 84-month surveys, and Ha Giang 60-, 72- and 96-month surveys. The BED assay measures the proportion of IgG that is anti-HIV antibody and uses a multi-subtype synthetic peptide antigen for detection of HIV-1 subtypes B, E, and D. For Ning Ming, frozen HIV-positive serum/plasma samples were available from all survey waves and BED testing was carried out by the laboratory of the Guangxi CDC in Nanning, China. For the Vietnamese sites, we had frozen HIV-positive serum/plasma samples only from three survey waves (48, 72, and 84 months) in Lang Son and from three survey waves (60, 72, and 96 months in Ha Giang Town). This was because samples from earlier waves were not frozen. BED testing of Vietnamese samples, except for those from the Ha Giang 96-month survey, was done at the Thai Ministry of Public Health-U.S. Centers for Disease Control and Prevention HIV/STI Laboratory in Nonthaburi, Thailand. BED testing for Ha Giang’s 96-month survey was done at the National Institute of Hygiene and Epidemiology in Hanoi.

### Ethical Review

The study was reviewed and approved by the institutional review boards (IRBs) of the following institutions: Guangxi Center for HIV/AIDS Prevention and Control, the National AIDS Standing Bureau of Vietnam, Lang Son and Ha Giang Provincial Health Services, Abt Associates Inc., and Beth Israel Medical Center, New York, NY. The BED testing of Vietnamese samples in Thailand was done under a non-research determination from CDC.

### Analysis

Data sets were prepared in EpiInfo, version 6.04 by staff of the Guangxi Center for HIV/AIDS Prevention and Control (for Ning Ming) and, over time in Vietnam by staff of the National AIDS Standing Bureau and Abt Associates. The data were analyzed at Abt Associates Inc. using SAS, Version 9.22 (SAS, Inc., Cary, NC). For all analyses, we considered a measure to be statistically significant if it had an estimated p-value of less than or equal to 0.05.

### Trends in HIV Risk Behaviors and HIV Prevalence

To analyze trends in risk behaviors and HIV prevalence, we used regression analysis to estimate a time trend for each behavior and HIV prevalence. Separate regressions were run for each project site. Tests of significance for trends used a linear time trend, adding covariates for specific geographic subunits. We also took into account the potential correlation generated by multiple responses from the same respondents over time, using a robust covariance matrix with clustering on the survey respondent. The robust covariance matrix will not affect the estimated parameters from a model assuming independent and identically distributed errors, but will change the estimated parameter covariance matrix [35]. To estimate the model for these outcomes, we used the SAS SURVEYLOGISTIC procedure and the CLUSTER command to cluster the error matrix on survey respondents [36]. The model assumes clustering among responses from the same participant but independence across survey participants.

### Comparative Analysis of HIV Prevalence Trends

To explore further the possible associations between the interventions and trends in HIV prevalence, we compared the trends in our Vietnam project sites to those from sentinel surveillance in other northern Vietnam provinces without similar interventions (Dien Bien, Lao Cai, Bac Giang, Cao Bang, and Phu Tho) and compared the trends in Ning Ming to those in Yunnan Province, China, which also lacked similar interventions during the period under study. For these comparisons, we used a difference-in-difference estimator [37] to calculate the trend in HIV prevalence within project sites and “difference out” the underlying trend in HIV prevalence occurring in comparison provinces.

Ha Giang began its intervention in 2003, while Lang Son began in 2002. In Dien Bien, sentinel surveillance data were available for 2004 forward. In Lao Cai, sentinel surveillance reported HIV prevalence at 3 to 6 percent in 2000 through 2003, but not less than 14 percent in subsequent years (18 to 22 percent in 2004 through 2006). The rise in HIV prevalence seems suspicious between 2003 and 2004, so we elected to use data from 2004 to 2009 for the Vietnam comparisons. Changing the time period would not substantially change the results. To determine the trend in the Vietnamese comparison sites, we used a weighted least-squares regression, with the weights equaling 1 divided by the square root of the number of tests in each site for each year (for details, see Model Specifications S1). The dependent variable was the proportion testing positive for that site in that year. Independent variables included dummy variables for each site, and a time trend for each site (equaling 0 in 2004 and 1 in 2009). To compute the change in prevalence across the five provinces, we used a simple average of the percent decline in prevalence over the time period 2004 to 2009.

We used similar estimation techniques for China to compare HIV prevalence trends among IDUs in Ning Ming with those in Yunnan Province for the period 2002–2007, based on data in a paper by Jia [38]. We estimated a weighted least-squares regression, with the weights equaling 1 divided by the square root of the number of tests for each year. The dependent variable was the proportion testing positive in that year. The independent variable was a time trend (equaling 0 in 2002 and 1 in 2007). In the comparison with Yunnan Province, we also added a time-squared trend to the model because it greatly improved model fit (for details, see Model Specifications S1).

In both countries, we ran similar regressions for our project sites. For each project site, we ran separate regressions that included dummy variables for the subsite (in Lang Son and Ning Ming), demographic variables that included marital status, ethnic minority status, age (dummy variable for 21–30), indicator for male, and a time trend. For each site, we ran a linear probability model. In China, we used a generalized estimating equation with repeated measures for each person. For Vietnam, we used an OLS regression.

### Trends in HIV Incidence

We employed two methods to estimate trends in HIV incidence: analysis of new injectors and BED testing. As described elsewhere [16], we estimated HIV incidence among new injectors, defined as individuals reporting injection histories of three years or less. To test the significance of a trend in estimated HIV incidence, we used a Generalized Estimating Equation [39] with a normal distribution and an identity link [35]. We estimated the GEE using the GENMOD procedure in SAS. In the GEE, the incidence was the dependent variable, the project site and survey wave were independent variables, and we allowed clustering when an individual appeared in more than one survey. The test of significance was based upon the z-statistic reported for the survey wave.

Our second method was based on BED testing, which can identify recent infections (approximately within the past six months) from samples that are HIV-positive on antibody tests [32]–[34]. BED results can be used to estimate HIV incidence from cross-sectional data [40].

Annual HIV incidence (*I*) was estimated based on the BED test results using the formula in the Calypte kit insert (*I* = ([365/w] N_inc_)/(N_neg_ + [365/w] N_inc_/2) where w, is the window or recency period, N_inc_ is the number of recent infections and N_neg_ is the number of HIV negatives in a survey wave. Using BED data from 190 seroconversion panels from Thailand [41], we determined the recency period (the mean period from initial seroconversion to an ODn of 0.8) as 152 days.

When specimens from all HIV-positives were not available for BED testing, the BED estimate was extrapolated by the factor: (# BED-recent/# of HIV-positive specimens available for testing). This assumes that the proportion of specimens that test positive for recent infection is the same as in those with available and missing specimens. Many of the calculated incidence rates were very close to or below zero. As a result, we computed confidence intervals based upon the Wilson score interval [42].

Adjustments to BED-based incidence estimates are advised because of the possibility of the test producing false-recent results. We selected the adjustment proposed by Welte [43]. This method adjusts well for variance in recency periods, among other factors proposed by McDougal [44] and Hargrove [45]. Such adjustments to very low incidence rates may generate negative values.

In addition to applying this adjustment, we examined survey responses for all participants with BED-recent results (linked through study ID numbers) on whether and when these individuals had been told that they had HIV infection and duration of any ARV treatment. Where linkage between names and study numbers was still available, we were able to check putative “recent” infections against registries of outpatient clinics providing ARV treatment. Any cases of long-term infection or ARV treatment initiated more than 6 months before the survey were excluded as recent infections for HIV incidence estimation.

## Results

The results are based on 5,695 responses to the cross-sectional surveys of IDUs: 2,125 in Ning Ming; 2,677 in Lang Son; and 1,060 in Ha Giang. Less than 5% of eligible respondents who were approached refused to participate.

Coverage of the interventions among IDUs was generally 60%–70% based on self-reported receipt of needles/syringes or pharmacy vouchers in the cross-sectional surveys (data not shown). This assumes that the survey samples were representative of the total IDU population in the sites. Over the course of the project, an average of 7,000–10,000 new needles/syringes were provided per month in each site, taking into account both direct distribution and redemption of pharmacy vouchers (Abt Associates Inc., unpublished data). The estimated numbers of IDUs were 1,700 in Ning Ming, 1,700 in Lang Son and 1,000 in Ha Giang, so that the program provided approximately 70 sterile syringes per IDU per year.

Analysis of demographic characteristics (data not shown) revealed that in all sites IDUs were predominantly males between 21 and 30 years old (although there was some tendency for the samples to age over time), and about two-thirds were single. Ethnic minorities (e.g., Nung or Tay in Vietnam; Zhuang, Dong, Miao, Yao, or Jing in China) contributed substantial percentages to these IDU samples: usually 70%–80% in Ning Ming, 45%–60% in Lang Son, and 20%–40% in Ha Giang.

Table 1 shows self-reported drug-related risk behaviors among IDUs. Regression-based linear time trends show that these measures fell in all sites over the course of the interventions and these reductions were generally significant.

**Table 1:** Drug-related HIV risk behaviors.

Site	Survey Wave	n	ReceptiveSharing, last6 months	Distributive Sharing,6 last months	Shared drug Solution,6 last months	Shared any injectionEquipment, 6 lastmonths
Ning Ming County, China	Baseline	290	47%	52%	41%	76%
	6 month	331	29%	25%	22%	53%
	12 month	303	22%	27%	18%	47%
	18 month	299	17%	17%	13%	31%
	24 month	209	9%	11%	8%	17%
	36 month	210	7%	8%	6%	14%
	48 month	238	14%	11%	6%	24%
	72 month	245	10%	10%	3%	15%
	p- value		<0.001	<0.001	<0.001	<0.001
Lang Son Province, Vietnam	Baseline	342	5%	6%	32%	47%
	6 month	340	5%	5%	39%	47%
	12 month	327	2%	1%	25%	46%
	18 month	335	3%	4%	32%	41%
	24 month	333	2%	1%	16%	30%
	48 month	336	1%	1%	23%	26%
	72 month	330	1%	2%	19%	25%
	84 month	334	1%	<1%	10%	12%
	p- value		0.004	<0.001	<0.001	<0.001
Ha Giang Town, Vietnam	Baseline^1^	100	23%	12%	56%	67%
	6 month^1^	100	6%	4%	16%	17%
	12 month^1^	98	14%	8%	39%	50%
	18 month^1^	98	2%	0%	41%	44%
	24 month	98	19%	20%	43%	59%
	36 month	99	5%	9%	39%	44%
	48 month	100	5%	3%	12%	16%
	60 month	100	1%	0%	20%	20%
	72 month	100	0%	0%	15%	16%
	96-month	167	10%	8%	28%	35%
	p- value		0.004	0.115	<0.001	<0.001

1 During this period, a Peer-Driven Intervention was being implemented; by the 24-month survey, the intervention had been changed to a peer outreach model.

Survey results regarding self-reported sexual risk behaviors (data not shown) reveal no clear trends. However, they indicate that from one-half to two-thirds of IDUs are sexually active and that they report low rates of condom use with regular partners but higher rates with casual or commercial partners. In most survey waves, less than 20% of IDUs reported patronizing sex workers.

**Figure 2:**
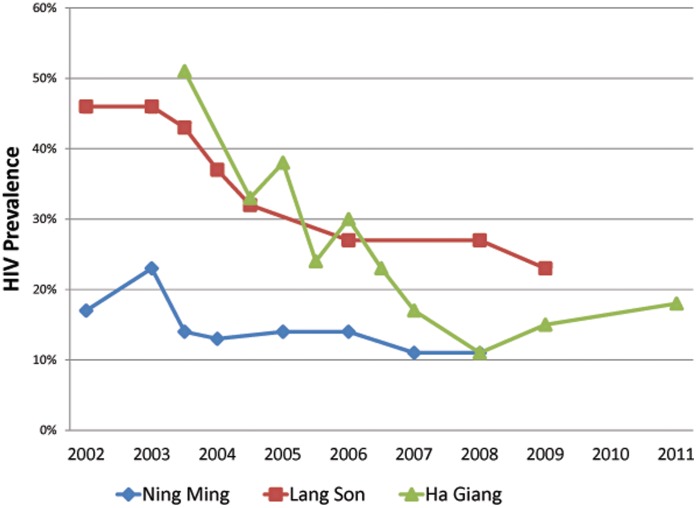
HIV prevalence trend among IDUs.

Table 2 and Figure 2 present the HIV prevalence trends, showing statistically significant reductions in the Cross-Border sites. While there were differences in the prevalence patterns across the comparison provinces in Northern Vietnam, with several increasing while others declined or were stable, the overall estimated change was a reduction of 1.2% with a standard error of 2.44% in these comparison provinces, which is statistically indistinguishable from no change (See Table S1 for details). In Lang Son, by contrast, the estimated change in HIV prevalence from 2004 to 2009 (i.e., from the 18- to 84-month surveys) was a reduction of 12.1% with a standard error of 3.0%. In Ha Giang (also from the 18- to 84-month surveys), the estimated change was a reduction of 32.1% with a standard error of 5.0%. When differencing out the underlying trend in other northern provinces, the prevalence reductions in both Lang Son (11.0%, p = 0.002) and Ha Giang (30.9%, p<0.001) from 2004 to 2009 are statistically different from zero and thus significantly larger than in the comparison provinces. In China, we estimated a 0.2% drop in HIV prevalence in Yunnan with a standard error of 1.5%, compared to a 4.4% drop in Ning Ming (i.e., from the 18–72 month surveys) with a standard error of 2.3% (see Table S2 for details). The standard errors are White heteroskedastic-consistent standard errors. The difference is statistically significant (4.1%, p = 0.019).

**Table 2:** HIV prevalence among IDUs: Cross-Sectional Surveys.

	Ning Ming County, China		Lang Son Province, Vietnam		Ha Giang Town, Vietnam	
Survey Wave	n	HIV Prevalence	n	HIV Prevalence	n	HIV Prevalence
Baseline	290	17%	342	46%	100	51%^1^
6 month	331	23%	340	46%	100	33%^1^
12 month	303	14%	327	43%	98	38%^1^
18 month	299	13%	335	37%	98	24%^1^
24 month	209	14%	333	32%	98	30%
36 month	210	14%		NA	99	23%
48 month	238	11%	336	27%	100	17%
60 month		NA		NA	100	11%
72 month	245	11%	330	27%	100	15%
84 month		NA	334	23%		NA
96 month		NA		NA	167	18%
p-value		0.003		<0.001		<0.001

1 During this period, a Peer-Driven Intervention was being implemented; by the 24-month survey, the intervention had been changed to a peer outreach model.

**Figure 3:**
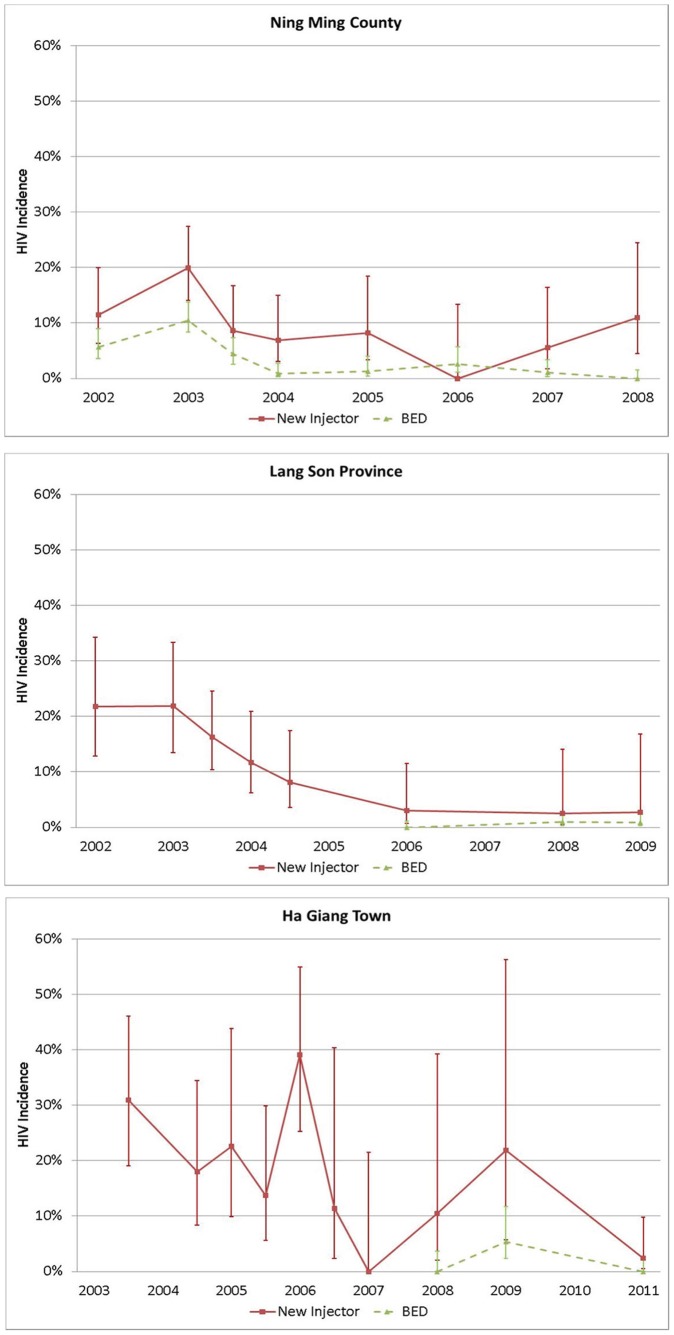
HIV Incidence Estimates by 2 Methods with 95% Confidence Intervals.

Table 3 and Figure 3 reveal reduced HIV incidence by both new injector and BED-based analysis. There are some apparent incidence increases among new injectors in the more recent survey waves, while the BED-based estimates reveal more consistently low incidence in these later waves. As shown in Figure 3, the 95% confidence intervals are substantially wider for the new injector-based estimates than for the BED-based estimates. This is in part due to the fact that there were far fewer new injectors in these later waves.

**Table 3:** HIV Incidence Estimates by 3 Methods.

	Ning Ming County, China			Lang Son Province, Vietnam			Ha Giang Town, Vietnam		
Survey Wave	Newinjector	BED	Welte Adjusted incidence	Newinjector	BED	Welte Adjusted incidence	Newinjector	BED	Welte Adjusted incidence
Baseline	0.12	0.06	0.03	0.22	NA	NA	0.31^1^	NA	NA
6 month	0.20	0.10	0.07	0.22	NA	NA	0.18^1^	NA	NA
12 month	0.09	0.04	0.02	0.16	NA	NA	0.23^1^	NA	NA
18 month	0.07	0.01	−0.01	0.12	NA	NA	0.14	NA	NA
24 month	0.08	0.01	−0.01	0.08	NA	NA	0.39	NA	NA
36 month	0.00	0.03	0.01	NA	NA	NA	0.11	NA	NA
48 month	0.06	0.01	−0.008	0.03	0.00	0.00	0.00	NA	NA
60 month	NA	NA	NA	NA	NA	NA	0.10	0.00	0.00
72 month	0.11	0.00	0.00	0.03	0.01	−0.01	0.22	0.05	0.03
84 month	NA	NA	NA	0.03	0.01	−0.004	NA	NA	NA
96 month	NA	NA	NA	NA	NA	NA	0.02	0.00	−0.04

1 During this period, a Peer-Driven Intervention was being implemented; by the 24-month survey, the intervention had been changed to a peer outreach model.

**Figure 4:**
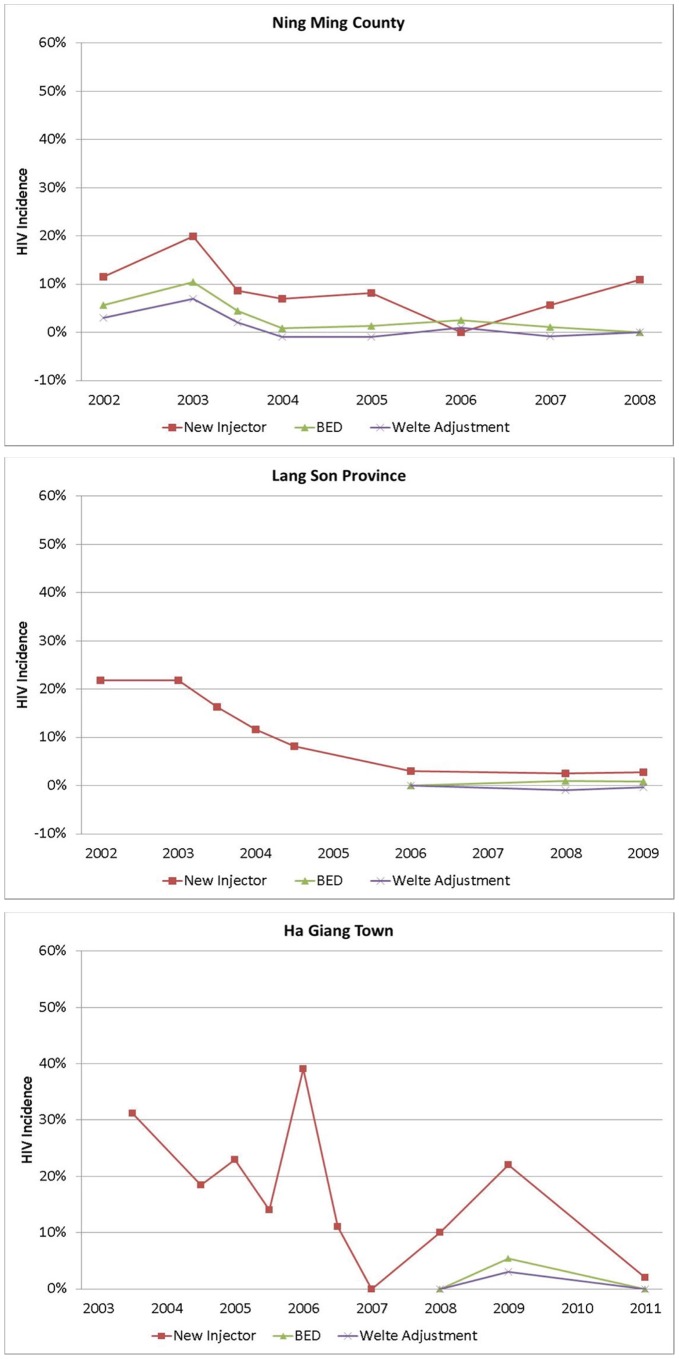
Incidence Estimates with Welte Adjustment.

To address potential problems with false-recent results in BED testing, we applied the Welte adjustment [43] and examined survey responses. Table 3 presents the BED-based incidence estimates following application of the Welte adjustment, revealing very similar trends in all sites. The unadjusted and adjusted BED-based incidence trends are shown together in Figure 4.

Survey responses confirmed the BED-based estimates for all but the Ha Giang 96-month survey. Of 41 BED-recent subjects from all sites and all waves, four reported having been previously told that they were HIV-infected. One individual who reported being told of his HIV infection about seven months prior to the survey in which his blood sample tested BED-recent was treated as a recent infection. However, all three individuals with “recent” infections based on BED testing of the 28 HIV-positive samples in the Ha Giang 96-month survey were found to have been on ARV treatment for 2–4 years. These determinations were based on information from questionnaire responses, outpatient clinic patient registries, and project peer educators. Thus, all three of these cases were considered false recents on BED testing, thus producing a 0% annual HIV incidence estimate for the Ha Giang 96-month survey.

## Discussion

The analysis of the Cross-Border Project data reveals consistent reductions in drug-related HIV risk behaviors, HIV prevalence, and HIV incidence among IDUs in the project sites in Northern Vietnam and Southern China. Since this is neither a randomized trial nor a quasi-experimental study but rather an ecological study based on cross-sectional data, we cannot draw firm conclusions that our interventions caused the positive trends we have observed. However, this problem is reduced in that the Cross-Border Project is implementing structural interventions, which are appropriately evaluated using community cross-sectional data. In addition, the consistency in trends across the primary outcomes strongly supports a positive intervention effect.

The Cross-Border findings suggest that peer-based interventions for IDUs that provide large numbers of needles/syringes by multiple means (direct distribution, pharmacy vouchers, and secondary exchange) without limits on numbers per contact can control HIV epidemics among IDUs. There have been similar findings from other places, for example, New York City where reductions in HIV incidence among IDUs followed large-scale implementation of peer outreach and needle/syringe programs [46].

The successful use of current IDUs as peer educators is also a noteworthy feature, as such PEs were demonstrated to have efficient access to and trust among the target population. Coverage of the Cross-Border interventions has been quite high (60%–70% of IDUs) in all sites but some reductions in coverage were observed in Ning Ming, China in the most recent cross-sectional surveys. This, and concomitant reductions in the average monthly numbers of needles/syringes provided, have probably resulted from reduced levels of funding for the interventions in Ning Ming. However, these changes have not yet occasioned any rebound in HIV prevalence or incidence among IDUs. This suggests that the interventions are truly structural in that they have changed behavioral norms in the IDU community and thus no longer need directly reach very high percentages of IDUs in order to maintain low levels of HIV risk behavior and associated HIV transmission. The success of such structural interventions depends greatly on building and maintaining community support and collaboration with police and other officials. The importance of such support has been noted in assessments of other needle/syringe programs in Vietnam [15] and has been successfully maintained over the long term by the Cross-Border Project [28].

There are several other issues and potential limitations regarding the design of the study and the methods of collecting and analyzing the primary outcome indicators that require discussion. It is possible that our survey samples are not fully representative of IDU populations in the sites. The use of snowball sampling methods may introduce sampling bias and dependence that may influence analytic results. However, we are unable to explore or adjust for such influences because, in part to protect participants’ confidentiality, we did not record which participants came from which snowball strings. Nevertheless, in studies of IDU populations, it is common practice to use targeted or snowball samples because these are often the best methods that are feasible. This point is supported in several extensive literature reviews [25], [47].

Self-reported behavioral data may be subject to biases. For example, the very low reported rates of needle/syringe sharing in the Vietnam sites from the very first survey waves may reflect responses based on social desirability. In fact, this seems likely given the high HIV incidence among new injectors in the early survey waves.

High risk sexual behaviors remain prevalent among IDUs, indicating a need to intensify interventions in this domain. Sexual partners of IDUs may be at particular risk. Indeed, cross-sectional surveys in this group, conducted to evaluate companion interventions for women at risk, revealed HIV prevalence of 2%–5% in Ning Ming and Ha Giang and a sharply higher 22% in Lang Son (Abt Associates, unpublished data). Cross-sectional surveys of sexual partners of IDUs in Hanoi reveal HIV prevalence between 9% and 14% [48].

In general, biological measures are more reliable than self-reported behavioral data for assessing the effectiveness of interventions. A strength of our evaluation is that we have collected biological measures. The Cross-Border data reveal major reductions in HIV prevalence among IDUs in Lang Son and Ha Giang and more modest declines in Ning Ming. Declining prevalence may reflect factors unrelated to intervention effects, such as deaths or mobility in the target or surveyed population. However, the declines in prevalence in our project sites are not likely due to underlying trends, since other provinces of northern Vietnam and in Yunnan Province, China, without such interventions did not experience similar declines in prevalence. These comparisons support the conclusion that the Cross-Border interventions have had a positive effect on declining HIV prevalence among IDUs.

HIV incidence is the most important indicator of effectiveness but few evaluations of HIV prevention interventions for IDUs have examined incidence trends. The Cross-Border project analysis reveals reduced HIV incidence in all sites although there are some differences between the new injector and BED-based incidence estimates. Incidence among new injectors declined sharply in all sites in the survey waves following full implementation of the interventions [16] but there appear to be some rebounds in later waves, especially in Ning Ming. We believe that these later results may be unreliable, particularly given the much lower incidence for these same waves based on BED testing. The new injector estimation method relies on an assumption that a new injector was HIV-negative at the time he initiated injection. While this may have been a safe assumption for the early survey waves when the HIV epidemic was growing sharply among IDUs, it may not be reliable for the later waves when the epidemic had become more mature and more sexual transmission had begun to occur. In these later waves, it is possible that an individual could already have been infected with HIV through sexual contact by the time he initiated injection, which would distort the incidence estimate upward. As a result, we are inclined to give more credence to the BED-based incidence estimates, especially for the later survey waves.

It is also important to recognize the possible problems with the BED-based estimates. The BED assay can be non-specific, particularly in African settings. False-recent BED test results can occur in persons with long-term infection and those on ARV treatment [49]–[55]. Such errors would tend to produce artificially inflated incidence estimates. However, in a recent study of BED performance in Thailand, where the epidemic is dominated by similar viral subtypes as found in northern Vietnam and southern China, very few false-recent cases were observed [34]. Application of adjustments for false-recency in that study reduced the incidence estimates to the conventional cohort-based observed incidence, suggesting that false-recency may be less problematic in Southeast Asia, or in the viral subtypes that predominate in this region. While debate continues on the most appropriate adjustment factor to apply for specific settings [56], we selected the Welte adjustment [43], as recommended by CDC and the Office of the Global AIDS Coordinator [57] and as used in the Thailand study [34]. Moreover, in our surveys, participant-level data were available allowing removal from analysis of cases that could generate false-recent results on the BED assay.

In any event, overestimation is not a problem for BED-based estimates in the later survey waves, which are already extremely low, except for the Ha Giang 96-month survey in which the three BED-based “recent” infections, if correctly identified, would have resulted in a sharp incidence increase because of the small numbers involved. As reported above, we found that these three cases were all false-recents on the BED test, resulting in a 0% annual incidence estimate for the Ha Giang 96-month survey.

Artificially high estimates in the earlier surveys could undermine the validity of the observed downward incidence trend. However, the BED and other assays to detect recent infections are ideally suited to longitudinal surveys such as this where the objective is to determine trends over time because assay-based biases that might be present at one time would likely persist at others, making it very likely that the downward trend observed in the BED-based incidence is real even if some overestimation existed. Moreover, the estimates from the new injector analysis, the survey responses regarding individuals’ duration of infection, and trends observed after the application of the Welte adjustment [43] all corroborate the higher incidence rates in the earlier survey waves and thus support the downward trend.

Combining the incidence trends from the new injector analysis and the BED testing, we find sharp declines in HIV incidence among IDUs in all sites. The only other study of IDUs in Vietnam that has measured HIV incidence was in Thai Nguyen Province, where an intervention without needle/syringe provision was being implemented. HIV incidence in a cohort study conducted there between 2005 and 2007 was 5% per year [58], higher than the rates found in the later Cross-Border survey waves.

Despite the limitations of the study, we conclude that the Cross-Border interventions have played an important role over an eight-year period in controlling HIV transmission among IDUs and, as a result, offer a model of HIV prevention for IDUs that should be considered for large-scale replication.

## Supporting Information

Model Specifications S1 Vietnam and China Prevalence comparisons.
(DOCX)

Table S1 Parameter estimates for WLS Regression in Vietnam Comparison Sites.
(DOCX)

Table S2 Parameter estimates for WLS Regression in China Comparison Site (Yunnan).
(DOCX)
