# Supplementary material for: Controlling HIV Epidemics among Injection Drug Users: Eight Years of Cross-Border HIV Prevention Interventions in Vietnam and China
Source: PLoS One. 2012 Aug 27;7(8):e43141. doi: 10.1371/journal.pone.0043141 (PMC3428343; doi:10.1371/journal.pone.0043141)
Supplement: Model Specifications S1 — Vietnam and China Prevalence comparisons. (DOCX) [file pone.0043141.s001.docx]

**Model specifications S1: Vietnam and China prevalence comparisons**

Model for Vietnam and Chinese comparison sites, estimated separately for each country:

$HIVPrevalence_{s,t}=\sum_{s=1}^{S} \left(\beta_{s}d_{s}+\gamma_{s}t_{s}+\delta_{s}t_{s}^{2}\right)+\varepsilon_{s,t}$, where

*s* denotes a province. Many provinces were appropriate for Vietnam, and only one was appropriate for China.

*d_s_* is an indicator that equals 1 if an observation is from province *s*,

$\beta_{s}$ is an intercept term for province *s*,

*t* is a time variable that ranges from 0 to 1. In Vietnam, 0 indicates 2004 and 1 indicates 2009; in China, 0 indicates 2002 and 1 indicates 2007.

$\gamma_{s}$is a linear time parameter for province *s*,

*t^2^* is the square of *t*, only appearing in the model for China,

$\delta_{s}$ is a squared time parameter for province *s*,

*HIVPrevalence_s,t_* is the HIV prevalence from province *s* at time *t*, and

$\varepsilon_{s,t}$ is a heteroskedastic error term specific to province *s* at time *t*. It is a weighted least squares error term, where the weights are proportional to the square root of the number of people tested in province *s* at time *t.*

For Vietnam, we attempted higher order time trends such as time squared and time cubed, but they were not statistically different from zero and were rejected from inclusion using an F-test. For China, we rejected including a time cubed term.

Model for Lang Son, Ha Giang, and Ning Ming, estimated separately for each project site

$HIVStatus_{i}=\alpha+\gamma t_{i}+\sum_{s=1}^{S} \beta_{s}d_{s,i}+\delta_{c}X_{c,i}+\varepsilon_{i}$, where

*i* denotes an individual,

*a* is an intercept term,

*s* denotes a commune from which the sample was taken(used only in the model for Lang Son),

$\beta_{s}$ is an intercept term for commune *s*,

*d_s,i_* is an indicator that equals 1 if an individual is from province *s*,

*t* is a time variable that ranges from 0 to 1. For Vietnam, 0 indicates 2004 and 1 indicates 2009; for China, 0 indicates 2002 and 1 indicates 2007.

$\gamma$ is a linear time parameter,

*X_c_* is a vector of covariates, denoted by *c*, to improve the efficiency of the estimated trend. These include whether a respondent was aged 21 to 30, male, married, never married, or an ethnic minority member,

$\delta_{c}$ are the parameters associated with *X_c_*

*HIVStatus_i_* is the HIV prevalence for individual *i*, and

$\varepsilon_{i}$ is an iid error term in Lang Son and Ha Giang, and an error term taking into account within-person correlations in Ning Ming.
