# Supplementary material for: Controlling HIV Epidemics among Injection Drug Users: Eight Years of Cross-Border HIV Prevention Interventions in Vietnam and China
Source: PLoS One. 2012 Aug 27;7(8):e43141. doi: 10.1371/journal.pone.0043141 (PMC3428343; doi:10.1371/journal.pone.0043141)
Supplement: Table S1 — Parameter estimates for WLS Regression in Vietnam Comparison Sites. (DOCX) [file pone.0043141.s002.docx]

**Table S1: Parameter estimates for WLS Regression in Vietnam Comparison Sites**

| **Variable** | **Estimate** | **StdErr** | **t** | **p-value** |
| --- | --- | --- | --- | --- |
| Intercept | 0.209 | 0.027 | 7.794 | <0.001 |
| Dien Bien | 0.133 | 0.040 | 3.343 | 0.001 |
| Bac Giang | -0.140 | 0.044 | -3.171 | 0.002 |
| Cao Bang | 0.036 | 0.035 | 1.026 | 0.305 |
| Phu Tho | 0.033 | 0.035 | 0.932 | 0.351 |
| t_0409LC | -0.050 | 0.043 | -1.170 | 0.242 |
| t_0409DB | 0.075 | 0.044 | 1.726 | 0.084 |
| t_0409BG | 0.095 | 0.053 | 1.794 | 0.073 |
| t_0409CB | -0.133 | 0.039 | -3.370 | 0.001 |
| t_0409PT | -0.046 | 0.039 | -1.185 | 0.236 |

Total tests = 24518

R-Square = 0.9093
