# Supplementary material for: Controlling HIV Epidemics among Injection Drug Users: Eight Years of Cross-Border HIV Prevention Interventions in Vietnam and China
Source: PLoS One. 2012 Aug 27;7(8):e43141. doi: 10.1371/journal.pone.0043141 (PMC3428343; doi:10.1371/journal.pone.0043141)
Supplement: Table S2 — Parameter estimates for WLS Regression in China Comparison Site (Yunnan). (DOCX) [file pone.0043141.s003.docx]

**Table S2: Parameter estimates for WLS Regression in China Comparison Site (Yunnan)**

| **Variable** | **Estimate** | **StdErr** | **t** | **p-value** |
| --- | --- | --- | --- | --- |
| Intercept | 0.278 | 0.014 | 20.270 | <0.001 |
| t_0207 | -0.002 | 0.023 | -0.107 | 0.914 |

Total tests = 16200

R-Square = 0.0029
